# A Cross-Sectional, Questionnaire-Based Survey on Air Infection Control among Romanian People

**DOI:** 10.3390/ijerph191912140

**Published:** 2022-09-25

**Authors:** Catalina Iulia Saveanu, Irina Nicoleta Zetu, Alexandra Scheuleac, Alexandra Ecaterina Saveanu, Cristian Liviu Romanec

**Affiliations:** 1 Department of Surgicals, Faculty of Dental Medicine, University of Medicine and Pharmacy Grigore T Popa, 700115 Iasi, Romania; 2 Faculty of Dental Medicine, University of Medicine and Pharmacy Grigore T Popa, 700115 Iasi, Romania

**Keywords:** infection control, precaution standard, air contaminants, aerosols

## Abstract

(1) Background: Infection control should be one of the main objectives in the comprehensive medical approach. (2) Methods: A cross-sectional study was conducted from June–July 2022. A questionnaire including 22 questions with multiple answers was applied online to 202 subjects selected at random. The questionnaire collected data on the subjects’ knowledge about protective measures against airborne infections. For this study, a descriptive statistic was performed. Pearson’s Chi-square test was used for data comparison. (3) Results: Only 61.39% (124) of the subjects consider that protective equipment is mandatory for the dental team, 40.10% (81) know to a very large extent and 44.06% (89) to a large extent that when talking, a respiratory infection can be transmitted. A rather large percentage of the subjects take into account the cost of the protective mask to a very large extent 39.60% (80). Only 30.20% (61) of the subjects would vaccinate to a very large extent and 24.75% (50) to a large extent against micro-organisms transmitted by air *p* > 0.05. (4) Conclusions: Most people know the aspects related to air decontamination, the priority being the promotion of control methods of airborne infections, and it is necessary to improve the level of knowledge on a large scale within the population.

## 1. Introduction

Respiratory infections are the leading cause of morbidity and mortality worldwide. Almost four million people die from acute respiratory infections each year, 98% of which from lower respiratory tract infections [1]. The main cause is the transmission of various microorganisms with pathogenic potential, different types of viruses or bacteria or the coexistence of several species [2,3,4,5]. Thus, in the case of microorganisms with epidemic or pandemic potential, special measures for infection control are necessary [6,7,8]. Infection control should be one of the main objectives in the comprehensive medical approach. 

In the dental office, the risk of transmission is higher due to the aerosol-generating operations [9,10,11,12]. Depending on the size, air pollutants can exist in several forms. Aerosols are particles between 5 and 50 millimicrons which are gradually deposited on surfaces for hours, fog with particles over 50 millimicrons are gradually deposited on surfaces for approximatively 5–15 min, droplets, larger than 50 millimicrons, which 30–40 cm around the oral cavity of the patient’s [13] dry cores are derived from moist airborne particles and contain potentially viable microorganisms and traces of secretions in the dry state. Dry nucleus from moist air particles containing potentially viable microorganisms and traces of dry secretions can remain suspended in the air indefinitely and can be transported on long distances [14].

Infection control must take into account the pathogenic characteristics of the microorganisms including modes of transmission, transmissibility, virulence and microbial load of the inoculum [15,16].

In modern medicine, infection prevention and control (IPC) measures must be a primary concern [17,18]. Thus, the measures to prevent the transmission of infection through the air are given by the adoption of Universal Precautions: pre-procedural oral rinses with antiseptic solutions (chlorhexidine gluconate, povidone-iodine, essential oils), perfect isolation of the operating field with a dike and adequate circulation measures of air: ventilation, air purification devices [19].

The lack of knowledge and attitudes about infection control increases the level of transmission of pathogenic agents, thus constituting a major threat to public health [20,21].

Effective information on the mode of transmission of airborne infections and the main preventive measures must represent a main goal and interdisciplinary collaboration should be the basis of health programs [19,22].

The aim of this study was to assess the level of knowledge regarding airborne infection control. The objectives of the study were: determining the level of information and awareness among the population regarding the transmission of infections through the air; assessing the level of knowledge and attitudes of the subjects in the context of airborne infection control; evaluation of the level of knowledge regarding the attitude of the subjects towards the use of protective masks and evaluation of the level of knowledge on the compliance of the subjects regarding the administration of vaccines for diseases transmitted through the air.

## 2. Materials and Methods

The evaluation of the level of knowledge was performed by the questionnaire method.

### 2.1. Setting and Participants

A cross-sectional study was conducted from June–July 2022. For the selection of the study group, we took into account the recommendations from the education and health guidelines. Practically, through our study we aimed to evaluate a level of knowledge, so according to the recommendations we applied the calculation formula. According to the calculation formula, Ref. [23] a minimum number of 196 subjects was representative for our study with 7% margin of error, 95% confidence level and 50% population proportion. The participants were represented by people over 18 years of age, male and female, from rural and urban areas. A total number of 202 subjects were included in the study sample. Participants sampling was volunteer based. The selected sample was representative for our area. The survey was sent to eligible participants using Google Docs. The study has alpha statistical power, α = <0.5, and with coefficient of reliability (or consistency) Cronbach’s Alpha = 0.482. 

### 2.2. The Survey

A questionnaire of knowledge about airborne infection control with multiple answers was applied to subjects selected at random. The questionnaire collected data on the subjects’ knowledge about protective measures against airborne infections. For this questionnaire, 22 questions were created. 

The questionnaire was reviewed for face validity by C.I.S., A.S. to identify key issues that may be relevant to subjects to assess its relevance and accuracy. During this process, 10 subjects completed the survey in full and then were interviewed by members of the research team to elicit their feedback and suggestions for improvement. The 10 students who completed the pilot-testing did not participate in the final survey and the responses collected during pilot-testing were not included in the final analysis. The answer variants that were not too clear were modified for better understanding. Then, the questionnaire was applied to the study group. Before completing the questionnaire, the participants gave their consent. Before the first question, explanations were given about the study in which the data about the study are specified and the ethics committee’s opinion is given. Then, there is the question about whether they agree to complete the questionnaire and then follows the completion of the questionnaire data. The questionnaire was applied in an open manner and was uploaded online on the Google Docs platform. The subjects were randomly selected by sharing a link on social media sites such as Facebook and WhatsApp Web.

### 2.3. Study Group

The selection of the study group was done following selection criteria in accordance with the ethical rules and good study practices. The study included randomly selected subjects from the Google Docs platform. In order to carry out this study, the informed consent issued by the ethics committee of the University of Medicine and Pharmacy Grigore T. Popa Iasi was obtained 199/03.06.2022. The inclusion criteria were: people over 18 years of age. The exclusion criteria were: people who did not agree to participate in the study. Eligible participants were those who agreed to complete the questionnaire after reading its contents. A total of 202 subjects completed the questionnaire. 

### 2.4. Demographic Characteristics

The collected demographic data were: age, gender, environment of origin. The study involved randomly selected subjects and later the distribution according to the environment of origin was found. 

### 2.5. Domain: Knowledge Data

The knowledge assessment focused on 19 multiple-choice questions about airborne infection control.

The 19 items (Q4–Q22) had multiple answer options between 3 and 5 options. The questions concerned:

Evaluation of the level of knowledge regarding the transmission of infection by air (Q4–Q9);

Assessment of the level of knowledge regarding the subjects’ attitude regarding airborne infection control (Q10–Q12); Evaluation of the level of knowledge regarding the subjects’ attitude regarding the use of protective masks (Q13–Q19); Evaluation of the level of knowledge regarding the compliance of the subjects related to the realization of vaccines for diseases transmitted by air (Q20–Q22). Likert Scale Response Options in Five-Point, level of Agreement was used [24].

### 2.6. Data Analysis 

For this study, a descriptive statistic was performed. Pearson’s Chi-square test was used for data comparison. The data were analyzed using IBM-SPSS version 26 (IBM, Armonk, NY, USA), with a *p* ≤ 0.05.

## 3. Results

### 3.1. Demographic Data

The study included 202 subjects with a mean age of 32.73 (±12.66) (min: max = 18:69) years. The distribution of subjects by gender was female–male = 59.9% (121): 40.1% (81).

### 3.2. Assessing the Level of Knowledge Regarding Airborne Infection Transmission

Only 61.39% (124) of the subjects consider that protective equipment is mandatory for the dental team. The rest of the subjects do not know the fact that the dental team must wear the mandatory protective equipment for all patients regardless of their health status (*p* > 0.05) (Table 1).

Moreover, the subjects do not know the ways of infection control by air, the form under which it can be transmitted. Thus, 28.71% (58) of the subjects answered that they consider the dental office to a small extent a source of contamination and 12.38% (25) to a very small extent and 30.20% (61) do not have any opinion (*p* > 0.05) (Table 1). Only 20.30% (41) of the subjects consider themselves protected in the dental office to a very large extent, and 40.59% (82) to a large extent (*p* > 0.05) (Table 1).

A percentage of 40.10% (81) of the subjects know to a very large extent and 44.06% (89) to a large extent that, a respiratory infection can be transmitted, when talking (*p* > 0.05) (Table 1).

The majority of the subjects do not know that a patient who is diagnosed with TB, who is not hospitalized and who is under treatment is not contagious (*p* > 0.05) (Table 1). More than half of the subjects do not know the fact that by ventilating the rooms the air is decontaminated. Only 15.84% (32) by subjects have correctly answering (*p* > 0.05) (Table 1).

### 3.3. Evaluation of the Level of Knowledge Regarding the Attitude of the Subjects in the Context of Airborne Infection Control

Regarding the importance of medical information on the presence of a disease with the potential for transmission through the air, 32.67% (66) of the subjects answered that this aspect is unimportant. Hand hygiene after sneezing or coughing is carried out by only 25.25% (51) of the subjects to a very high extent and by 48.02% (97) to a large extent. Mouthwash is used to a very large extent by 14.85% (30) of the subjects and by a large extent by 30.69% (62) (*p* > 0.05) (Table 2).

### 3.4. Evaluation of the Level of Knowledge Regarding the Attitude of the Subjects towards the Use of Protective Masks

The distribution of the responses regarding the importance of using protective masks highlighted the fact that only for 45.05% (91) of the subjects, this is at maximum (*p* > 0.05) (Table 3).

A rather large percentage of the subjects take into account the cost of the protective mask to a very large extent 39.60% (80) and to a large extent 16.34% (33) (*p* > 0.05) (Table 3).

**Figure 1:**
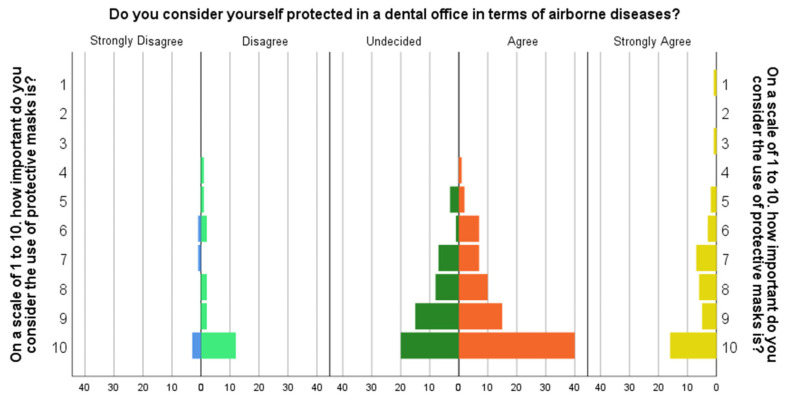
Subjects Pyramid Count “On a scale of 1 to 10. how important do you consider the use of protective masks is?” by “Do you consider yourself protected in a dental office in terms of airborne diseases?” The colors code is: Blue—Strongly Disagree; Light green—Disagree; Dark green—undecided; Orange—Agree, Yellow—Strongly Agree.

Among the most subjects who considered that wearing a protective mask is very efficient, a little over 15% of the subjects consider themselves protected in the dental office (Figure 1).

Regarding the filtering capacity of the protective masks, 30.69% (62) are indifferent, 16.34% (33) taking into account very much and 39.60% (80) to a great extent. On the other hand, the subjects are interested in the filtration capacity of the masks, 24.5% (50) to a very large extent and 31.68 (64) to a large extent (*p* > 0.05) (Table 3).

Only 13.37% (27) know the correct protection time for a surgical mask, the differences being statistically significant depending on gender *p* = 0.060 and depending on environment (*p* = 0.190) (Table 3).

Very few of the subjects, 21.29% (43), consider themselves subject to a risk if the obligation to wear protective masks is eliminated.

Only 35.15% consider to a very great extent and 43.56% (88) to a great extent that if they are diagnosed with a respiratory infection, they must necessarily wear a protective mask (*p* > 0.05) (Table 3).

**Figure 2:**
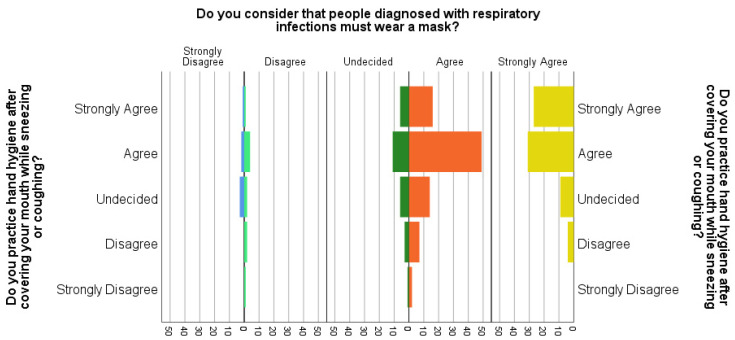
Subjects Pyramid Count “Do you practice hand hygiene after covering your mouth while sneezing or coughing?” by “Do you consider that people diagnosed with respiratory infections must wear a mask?” The colors code is: Blue—trongly Disagree; Light green—Disagree; Dark green—undecided; Orange—Agree, Yellow—Strongly Agree.

Additionally, a little over 20% of the subjects practice hand hygiene after sneezing or coughing, although they are aware that if you have an acute respiratory disease, you must wear a mask (Figure 2).

### 3.5. Evaluation of the Level of Knowledge on the Compliance of the Subjects Regarding the Administration of Vaccines for Diseases Transmitted through the Air

Only 30.20% (61) of the subjects would vaccinate to a very large extent and 24.75% (50) to a large extent against microorganisms transmitted by air (*p* > 0.05) (Table 4).

**Figure 3:**
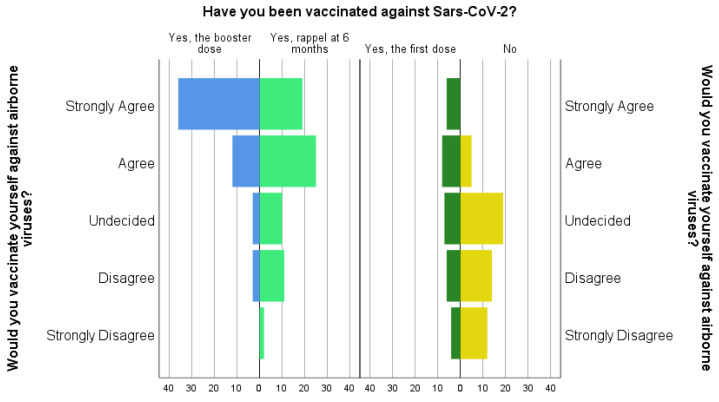
Subjects Pyramid Count “Would you vaccinate yourself against airborne viruses?” by “Have you been vaccinated against SARS-CoV-2?” The colors code is: Blue—Yes, the booster dose; Light green—Yes, rappel at 6 months; Dark green—Yes, the first dose; Yellow—No.

Regarding the vaccine against SARS-CoV-2, 24.75% (50) did not get vaccinated, 15.35% (31) took the first dose, 33.17% (67) had a booster at 6 months and 26.73 % (54) also had the booster dose, the results being significant from a statistical point of view (*p* = 0.010) (Table 4, Figure 3). 

Although the TB vaccine is mandatory in Romania, 36.14% (73) do not know if they have had it, 35.15% (78) declared that they did not have it and 28.71% (58) declared that they did have it (*p* > 0.05) (Table 4).

## 4. Discussion

Based on the analysis of our results, we can find a majority participation in the study of people aged between 18 and 40 years. This aspect could be justified based on the place where the study was carried out, namely the online environment, the Google Docs platform. Younger people show a much greater interest in online platforms. Additionally, the people from the urban environment seemed more interested in contributing to the realization of the study. Airborne infections represent a category of diseases with a very high level of importance.

### 4.1. Assessment of the Level of Knowledge Regarding the Transmission of Infection by Air

Aerosols represent a means of contamination in dental offices. Due to the fact that there is a direct correlation between respiratory system infections of dental staff and the concentration of aerosols in the office, patients must declare any respiratory disease [25]. Contaminant particles in the air can remain on surfaces or move according to the air flow [26,27].

Thus, air decontamination is a very important measure in infection control [28]. The dental office must be naturally ventilated for at least 15 min after each patient and the UV lamp activated according to its instructions for use [1].

A similar study conducted by King Abdulaziz University in Saudi Arabia highlights that most participants agreed that during treatment dentists should wear gloves (98.2%), protective mask (96.9%) should replace gloves after receiving a phone call (80%) and not use them for another patient (89.8%) [29].

If a patient has a symptomatic respiratory disease, it is necessary to approach a special protocol that includes disposable impermeable gowns, FFP2 or FFP3 protective mask [30,31,32].

Microorganisms are known to remain in the air for variable periods of time. If a person with an airborne infection has been treated, rooms where aerosol-generating procedures were performed should be ventilated for 1–3 h, unless operating under negative pressure. In the absence of another decontamination measure, air purifiers with HEPA filters, airing the premises is an effective measure. In situations where the ventilation system operates in a closed circuit, it is necessary to use high efficiency air filters (HEPA) for the recirculated air [33,34,35,36,37].

### 4.2. Assessment of the Level of Knowledge Regarding the Attitude of the Subjects Regarding the Control of Airborne Infection

The association between hand hygiene and infection prevention has been well known since the 19th century thanks to Semmelweis’ studies [38]. A very large number of infectious particles are spread through the sneeze or cough of an infected person. 

Recent studies demonstrate the effectiveness of mouthwash against the COVID-19 virus [39,40,41,42,43,44]. Regarding the results of the study, 45.5% of the subjects claim that they use mouthwash daily to a great extent and to a very great extent. More awareness programs are needed on the benefits of mouthwashes, which are effective not only in preventing and treating oral diseases, but also in preventing airborne infections.

### 4.3. Assessing the Level of Knowledge Regarding the Attitude of Subjects towards the Use of Protective Masks

The preventive effect of wearing masks in Asia has been shown to be greater than in Western countries especially against influenza viruses [45]. The wearing of the protective mask contributed to infection control [46].

Price should not be a determining factor in choosing a protective mask. Subjects must place much greater importance on the materials the masks are made of, the number of layers of the mask and the filters present. Our study suggests that price is an important decision factor in choosing protective masks. 

The air filtering capacity is the most important feature that defines the effective-ness of a mask. The filtering capacity of a surgical mask is environed by 90% depending on the manufacturer, and the filtering capacity of respiratory protective masks is between 80 and 99% [47,48,49,50].

Surgical masks have not been found to affect breathing capacity in a healthy person. In general, textile masks have a filtration rate between 49% and 86% for exhaled particles of 0.02 µm but changing masks at intervals of less than 4 h can be an effective preventive measure [51].

### 4.4. Assessing of the Level of Knowledge Regarding the Compliance of the Subjects According the Administration of Vaccines for Airborne Diseases

Vaccines are another topic addressed in the study. This protection method is among the most effective and easiest to achieve.

Immunization against airborne diseases is currently a priority at the international level; thus, vaccines against tuberculosis (BCG), diphtheria, whooping cough, pneumonia, seasonal flu, measles, rubella and mumps are administered [52].

Currently, SARS-CoV-2 represents a worldwide problem, and the issue of vaccination is very often addressed in specialized studies [53,54,55]. The results of several studies highlight the lack of information of the subjects regarding this immunization measure and the risk to which they are subjected [56,57]. 

Limitations of this study mattered in the fact that this was a cross-sectional questionnaire study.

Another limitation of this study was given by the small number of participants, unequal gender distribution, our study having more male subjects, random selection of subjects and lack of assessment of bias.

Additionally, this study had a large variability related to age groups, the majority of respondents being young and the lack of correlation with the social and financial level of the individual.

Additional studies were considered necessary to identify research opportunities at the general level of education, but also at the level of knowledge in dental practice, to increase awareness of infection control.

## 5. Conclusions

Within the limits of this study, we can emphasize the following conclusions.

Regarding the knowledge about the ways of transmission of airborne infections in the dental office, the majority of participants considered the dental office a safe environment and therefore considered it unlikely that they would contact an airborne infection in the dental office.

Most of the subjects consider that it is important to inform the dentist when they suffer from an infection that can be transmitted by air.

Most subjects know their responsibilities when they are in a dental office, but they also know the duties of the medical staff.

The subjects of our study believe to a very large extent that face masks are effective in protecting against airborne infections and feel more vulnerable in contacting infections following the removal of the mandatory mask wearing measure in crowded spaces

According to the results, when choosing a protective mask, the subjects take into account the cost of the masks to the greatest extent, then the breathing capacity and finally the air filtering capacity.

Regarding vaccination, most of the participants in the study claim that they agree with this method of immunization; therefore, they would agree to get vaccinated against airborne infections.

From another perspective, less than half of the subjects claim to be vaccinated against tuberculosis.

Even if tuberculosis still represents a serious health problem worldwide as well as in our country, the subjects of our study are not very well informed about this condition, a large part of the participants considering that people diagnosed with tuberculosis, under treatment, are contagious.

The majority of the subjects of this study consider hand hygiene to be an important practice, while the use of mouthwash is considered by them to be a less used protection method.

Most people know the aspects related to air decontamination, the priority being the promotion of control methods of airborne infections.

It is considered necessary to improve the level of knowledge on a large scale within the population, so that people can assume, adopt and apply preventive measures correctly, in this way managing to protect both themselves and those around them.

## Figures and Tables

**Table 1:** Distribution of responses regarding control of airborne infection.

Question	Total		Gender				*p*	*χ* ^2^	Environment				*p*	*χ* ^2^
	*N*	%	Female		Male				Rural		Urban			
			*N*	%	*N*	%			*N*	%	*N*	%		
Q 4 = Do you believe that the dental team is required to wear protective equipment even if you are healthy?														
Strongly Agree	124	61.39	74	36.63	50	24.75	0.955 ^a, b^	0.67	49	24.26	75	37.13	0.271 ^a, b^	5.16
Agree	60	29.70	35	17.33	25	12.38			22	10.89	38	18.81		
Undecided	13	6.44	9	4.46	4	1.98			7	3.47	6	2.97		
Disagree	2	0.99	1	0.50	1	0.50			2	0.99	0	0.00		
Strongly Disagree	3	1.49	2	0.99	1	0.50			2	0.99	1	0.50		
Q 5 = Do you consider the dentist’s office a source of airborne infection?														
Strongly Agree	21	10.40	12	5.94	9	4.46	0.847	1.38	5	2.48	16	7.92	0.570	2.93
Agree	37	18.32	23	11.39	14	6.93			15	7.43	22	10.89		
Undecided	61	30.20	37	18.32	24	11.88			27	13.37	34	16.83		
Disagree	58	28.71	32	15.84	26	12.87			24	11.88	34	16.83		
Strongly Disagree	25	12.38	17	8.42	8	3.96			11	5.45	14	6.93		
Q 6 = Do you consider yourself protected in a dental office in terms of airborne diseases?														
Strongly Agree	41	20.30	21	10.40	20	9.90	0.493 ^a^	3.40	15	7.43	26	12.87	0.309 ^a^	4.80
Agree	82	40.59	47	23.27	35	17.33			29	14.36	53	26.24		
Undecided	54	26.73	37	18.32	17	8.42			27	13.37	27	13.37		
Disagree	20	9.90	13	6.44	7	3.47			10	4.95	10	4.95		
Strongly Disagree	5	2.48	3	1.49	2	0.99			1	0.50	4	1.98		
Q7 = Did you know that respiratory infections can be transmitted when you talk?														
Strongly Agree	81	40.10	47	23.27	34	16.83	0.677 ^a^	2.32	24	11.88	57	28.22	0.055 ^a^	9.25
Agree	89	44.06	51	25.25	38	18.81			42	20.79	47	23.27		
Undecided	22	10.89	16	7.92	6	2.97			9	4.455	13	6.436		
Disagree	7	3.47	5	2.48	2	0.99			5	2.475	2	0.99		
Strongly Disagree	3	1.49	2	0.99	1	0.495			2	0.99	1	0.495		
Q 8 = Do you think that an outpatient diagnosed with TB under treatment is contagious?														
Strongly Agree	51	25.25	29	14.36	22	10.89	0.413 ^a^	3.95	16	7.921	35	17.33	0.370 ^a^	4.28
Agree	76	37.62	42	20.79	34	16.83			32	15.84	44	21.78		
Undecided	50	24.75	31	15.35	19	9.406			23	11.39	27	13.37		
Disagree	19	9.41	15	7.43	4	1.98			7	3.465	12	5.941		
Strongly Disagree	6	2.97	4	1.98	2	0.99			4	1.98	2	0.99		
Q 9 = Do you think that the airing of the space where you usually sit is sufficient for air decontamination?														
Strongly Agree	32	15.84	17	8.42	15	7.43	0.799 ^a^	1.653	11	5.45	21	10.40	0.390 ^a^	4.119
Agree	66	32.67	42	20.79	24	11.88			29	14.36	37	18.32		
Undecided	63	31.19	36	17.82	27	13.37			21	10.40	42	20.79		
Disagree	37	18.32	23	11.39	14	6.93			19	9.41	18	8.91		
Strongly Disagree	4	1.98	3	1.49	1	0.50			2	0.99	2	0.99		

^a, b^ Results are based on nonempty rows and columns in each innermost subtable. *N* = count; *χ*^2^ = chi-square test.

**Table 2:** Distribution of responses regarding the attitude of subjects toward airborne infection control.

Question	Total		Gender				*p*	*χ* ^2^	Environment				*p*	*χ* ^2^
	*N*	%	Female		Male				Rural		Urban			
			*N*	%	*N*	%			*N*	%	*N*	%		
Q 10 = Do you think it is important to inform the dentist when you suffer from an infection transmitted by air?														
Unimportant	2	0.99	1	0.50	1	0.50	0.799 ^a, b^	1.01	1	0.50	1	0.50	0.868 ^a, b^	0.72
Slightly Important	12	5.94	6	2.97	6	2.97			4	1.98	8	3.96		
Important	66	32.67	38	18.81	28	13.86			29	14.36	37	18.32		
Very Important	122	60.40	76	37.62	46	22.77			48	23.76	74	36.63		
Q 11 = Do you practice hand hygiene after covering your mouth while sneezing or coughing?														
Strongly Agree	51	25.25	32	15.84	19	9.41	0.953 ^a^	0.69	23	11.39	28	13.86	0.158 ^a^	6.61
Agree	97	48.02	59	29.21	38	18.81			34	16.83	63	31.19		
Undecided	34	16.83	19	9.41	15	7.43			18	8.91	16	7.92		
Disagree	16	7.92	9	4.46	7	3.47			7	3.47	9	4.46		
Strongly Disagree	4	1.98	2	0.99	2	0.99			0	0.00	4	1.98		
Q 12 = Do you use mouthwash on a daily basis?														
Strongly Agree	30	14.85	22	10.89	8	3.96	0.111	7.51	9	4.46	21	10.40	0.318	4.71
Agree	62	30.69	34	16.83	28	13.86			25	12.38	37	18.32		
Undecided	46	22.77	22	10.89	24	11.88			16	7.92	30	14.85		
Disagree	46	22.77	32	15.84	14	6.93			22	10.89	24	11.88		
Strongly Disagree	18	8.91	11	5.45	7	3.47			10	4.95	8	3.96		

^a, b^ Results are based on nonempty rows and columns in each innermost subtable. *N* = count; *χ*^2^ = chi-square test.

**Table 3:** Distribution of responses regarding the subjects’ attitude towards the use of protective masks.

Question	Total		Gender				*p*	*χ* ^2^	Environment				*p*	*χ* ^2^
	*N*	%	Female		Male				Rural		Urban			
			*N*	%	*N*	%			*N*	%	*N*	%		
Q 13 = On a scale of 1 to 10. how important do you consider the use of protective masks is?														
1	1	0.50	1	0.50	0	0.00	0.155 ^a, b^	11.91	0	0.00	1	0.50	0.332 ^a, b^	9.12
2	0	0.00	0	0.00	0	0.00			0	0.00	0	0.00		
3	1	0.50	0	0.00	1	0.50			1	0.50	0	0.00		
4	2	0.99	0	0.00	2	0.99			0	0.00	2	0.99		
5	8	3.96	6	2.97	2	0.99			6	2.97	2	0.99		
6	14	6.93	9	4.46	5	2.48			6	2.97	8	3.96		
7	22	10.89	10	4.95	12	5.94			9	4.46	13	6.44		
8	26	12.87	12	5.94	14	6.93			8	3.96	18	8.91		
9	37	18.32	22	10.89	15	7.43			17	8.42	20	9.90		
10	91	45.05	61	30.20	30	14.85			35	17.33	56	27.72		
Q 14 = When purchasing a mask, do you take into account its cost?														
Strongly Agree	33	16.34	19	9.41	14	6.93	0.735 ^a^	2.00	17	8.42	16	7.92	0.194 ^a^	6.06
Agree	80	39.60	46	22.77	34	16.83			30	14.85	50	24.75		
Undecided	58	28.71	39	19.31	19	9.41			26	12.87	32	15.84		
Disagree	23	11.39	13	6.44	10	4.95			5	2.48	18	8.91		
Strongly Disagree	8	3.96	4	1.98	4	1.98			4	1.98	4	1.98		
Q 15 = When choosing a protective mask, do you take its filtering capacity into account?														
Strongly Agree	33	16.34	24	11.88	9	4.46	0.498	3.37	11	5.45	22	10.89	0.356	4.39
Agree	70	34.65	39	19.31	31	15.35			31	15.35	39	19.31		
Undecided	62	30.69	36	17.82	26	12.87			24	11.88	38	18.81		
Disagree	27	13.37	17	8.42	10	4.95			14	6.93	13	6.44		
Strongly Disagree	10	4.95	5	2.48	5	2.48			2	0.99	8	3.96		
Q 16 = When choosing a mask, do you take its breathing capacity into account?														
Strongly Agree	50	24.75	39	19.31	11	5.45	0.031 ^a,^ *	10.62	17	8.42	33	16.34	0.589 ^a^	2.82
Agree	64	31.68	37	18.32	27	13.37			26	12.87	38	18.81		
Undecided	55	27.23	28	13.86	27	13.37			25	12.38	30	14.85		
Disagree	25	12.38	14	6.93	11	5.45			12	5.94	13	6.44		
Strongly Disagree	8	3.96	3	1.49	5	2.48			2	0.99	6	2.97		
Q 17 = How long do you think that a surgical mask offers you protection against the transmission of infection through the air?														
30 min	27	13.37	16	7.92	11	5.45	0.060	10.59	14	6.93	13	6.44	0.190	7.44
1 h	37	18.32	23	11.39	14	6.93			20	9.90	17	8.42		
2 h	46	22.77	21	10.40	25	12.38			13	6.44	33	16.34		
3 h	38	18.81	29	14.36	9	4.46			14	6.93	24	11.88		
4 h	47	23.27	26	12.87	21	10.40			18	8.91	29	14.36		
All the time unless it is damaged	7	3.47	6	2.97	1	0.50			3	1.49	4	1.98		
Q 18 = Do you think the removal of protective masks from crowded spaces is putting you at risk?														
Strongly Agree	43	21.29	23	11.39	20	9.90	0.135 ^a^	7.02	19	9.41	24	11.88	0.347 ^a^	4.46
Agree	87	43.07	53	26.24	34	16.83			40	19.80	47	23.27		
Undecided	48	23.76	33	16.34	15	7.43			15	7.43	33	16.34		
Disagree	18	8.91	7	3.47	11	5.45			5	2.48	13	6.44		
Strongly Disagree	6	2.97	5	2.48	1	0.50			3	1.49	3	1.49		
Q 19 = Do you consider that people diagnosed with respiratory infections must wear a mask?														
Strongly Agree	71	35.15	44	21.78	27	13.37	0.693 ^a^	0.693 ^a^	27	13.37	44	21.78	0.714 ^a^	0.714 ^a^
Agree	88	43.56	48	23.76	40	19.80			36	17.82	52	25.74		
Undecided	27	13.37	18	8.91	9	4.46			10	4.95	17	8.42		
Disagree	10	4.95	7	3.47	3	1.49			6	2.97	4	1.98		
Strongly Disagree	6	2.97	4	1.98	2	0.99			3	1.49	3	1.49		

^a, b^ Results are based on nonempty rows and columns in each innermost subtable. * *N* = count; *p* * = significance level; *χ*^2^ = chi-square test.

**Table 4:** The distribution of answers concerning the compliance of the subjects regarding the administration of vaccines for diseases transmitted through the air.

Question	Total		Gender				*p*	*χ* ^2^	Environment				*p*	*χ* ^2^
	*N*	%	Female		Male				Rural		Urban			
			*N*	%	*N*	%			*N*	%	*N*	%		
Q 20 = Would you vaccinate yourself against airborne viruses?														
Strongly Agree	61	30.20	39	19.31	22	10.89	0.412	3.96	22	10.89	39	19.31	0.519	3.24
Agree	50	24.75	27	13.37	23	11.39			22	10.89	28	13.86		
Undecided	39	19.31	22	10.89	17	8.42			16	7.92	23	11.39		
Disagree	34	16.83	19	9.41	15	7.43			17	8.42	17	8.42		
Strongly Disagree	18	8.91	14	6.93	4	1.98			5	2.48	13	6.44		
Q 21 = Have you been vaccinated against Sars-CoV-2?														
No	50	24.75	33	16.34	17	8.42	0.615	1.80	18	8.91	32	15.84	0.010 *	11.29
Yes, the first dose	31	15.35	20	9.90	11	5.45			21	10.40	10	4.95		
Yes, rappel at 6 months	67	33.17	37	18.32	30	14.85			23	11.39	44	21.78		
Yes, the booster dose	54	26.73	31	15.35	23	11.39			20	9.90	34	16.83		
Q 22 = Are you vaccinated against tuberculosis?														
Yes	58	28.71	34	16.83	24	11.88	0.972	0.06	19	9.41	39	19.31	0.278	2.56
No	71	35.15	43	21.29	28	13.86			29	14.36	42	20.79		
I do not know	73	36.14	44	21.78	29	14.36			34	16.83	39	19.31		

* *N* = count; *p* * = significance level; *χ*^2^ = chi-square test.

## Data Availability

Not applicable. For more data, you can contact the corresponding authors.
